# Primary bedaquiline resistance in Karakalpakstan, Uzbekistan

**DOI:** 10.5588/ijtld.22.0536

**Published:** 2023-05-01

**Authors:** S. Moe, M. L. Rekart, D. Hernandez, A. Sholpan, A. Ismailov, M. Oluya, A. Bayniyazova, T. Zinaida, P. Nargiza, C. Gomez-Restrepo, N. Sitali, A. Sinha

**Affiliations:** 1 Medecins Sans Frontières, Karakalpakstan, Uzbekistan; 2 Republican Center of Tuberculosis and Pulmonology, Nukus, Uzbekistan; 3 Republican Specialized Scientific and Practical Medical Center of Tuberculosis and Pulmonology, Tashkent, Uzbekistan; 4 Medecins Sans Frontières (MSF), Tashkent, Uzbekistan; 5 MSF, Berlin, Germany; 6 MSF, London, UK

**Keywords:** XDR-TB, DR-TB treatment, Class A TB drugs, BDQ-resistant TB, Central Asia

## Abstract

**BACKGROUND:**

Bedaquiline (BDQ) is widely used in the treatment of rifampicin-resistant TB (RR-TB). However, resistance to BDQ is now emerging. There are no standardised regimens for BDQ-resistant TB. This study aims to share experience in managing primary BDQ-resistant TB.

**METHODS:**

We performed a retrospective study of patients treated for RR-TB in Karakalpakstan, Uzbekistan, from January 2017 to March 2022. We identified patients with resistance to BDQ with no history of BDQ exposure. We describe baseline characteristics, treatment and follow-up of these patients.

**RESULTS:**

Twelve of the 1,930 patients (0.6%) had baseline samples resistant to BDQ with no history of BDQ exposure, 75% (9/12) of whom had been previously treated for TB. Ten (83.3%) were resistant to fluoroquinolones; respectively 66% and 50% had culture conversion by Month 3 and Month 6. The interim treatment outcomes were as follows: unfavourable treatment outcomes (3/12, 25%), favourable outcomes (2/12, 17%); the remaining seven (58%) were continuing treatment.

**CONCLUSIONS:**

A large proportion of the cases had previously been treated for TB and had TB resistant to quinolone. Both patients who had not experienced culture conversion by Month 3 had an unfavourable treatment outcome. Therefore, we recommend monthly monitoring of culture status for patients on treatment regimens for BDQ resistance.

## Abstract (fr)

**CONTEXTE:**

La bédaquiline (BDQ) est très utilisée dans le traitement de la TB résistante à la rifampicine (RR-TB). Cependant, une résistance à la BDQ est en train d’émerger. Il n’existe aucun schéma thérapeutique standardisé pour la TB résistante à la BDQ. Cette étude vise donc à partager l’expérience de la prise en charge de la TB primaire résistante à la BDQ.

**MÉTHODES:**

Nous avons réalisé une étude rétrospective auprès de patients traites pour RR-TB a Karakalpakstan, Ouzbekistan, de janvier 2017 à mars 2022. Nous avons identifié les patients présentant une résistance à la BDQ sans antécédents d’exposition à la BDQ. Nous décrivons les caractéristiques à l’inclusion, le traitement et le suivi de ces patients.

**RÉSULTATS:**

Douze des 1 930 patients (0,6%) présentaient, a l’inclusion, des échantillons résistants a la BDQ sans antécédents d’exposition a la BDQ, dont 75% (9/12) avaient déjà été traites pour une TB. Dix d’entre eux (83,3%) présentaient une résistance aux fluoroquinolones ; 66% et 50% respectivement ont été associes a une conversion des cultures au Mois 3 et au Mois 6. L’issue intermédiaire du traitement était la suivante : issue défavorable (3/12, 25%), issue favorable (2/12, 17%) et les sept restants (58%) étaient toujours sous traitement.

**CONCLUSIONS:**

Une grande partie des cas avaient déjà été traités pour une TB et présentaient une TB résistante aux quinolones. Les deux patients sans conversion des cultures au Mois 3 ont connu une issue thérapeutique défavorable. Par conséquent, nous recommandons une surveillance mensuelle de l’état des cultures pour les patients sous traitement dans le cadre d’une résistance à la BDQ.

The emergence and growth of multidrug/rifampicin-resistant TB (MDR/RR-TB) represents a major public health threat. In 2020, the WHO estimated 132,222 incident cases of MDR/RR-TB worldwide, plus 25,681 cases of pre-extensively drug-resistant (pre-XDR-TB) and extensively drug-resistant TB (XDR-TB),^1^,^2^ many of whom were not notified. Treatment of drug-resistant TB (DR-TB) is complicated, requiring 6–9 months of four or more drugs for MDR/RR-TB and pre-XDR-TB, and longer individualised regimens with additional drugs for up to 20 months for XDR-TB.^3^ The emergency of resistance to TB drugs has a significant impact on the final treatment outcome.^4^

Bedaquiline (BDQ) is a potent Class A drug with a novel mechanism of action leading to bactericidal and sterilising activity against *Mycobacterium tuberculosis* (MTB).^3^ BDQ-containing regimens significantly increase the rate of culture conversion at 24 weeks,^5^,^6^ and phase III clinical trials using BDQ as a backbone of the treatment regimen have proven that BDQ-containing regimens have a higher treatment success rate among MDR/RR-TB patients.^7^

The current known mechanisms of resistance to BDQ include 1) target-based resistance mutations in the *atpE* gene; and 2) mutations in *Rv0678* and pepQ (*Rv2535c*) that confer non-target-based resistance to BDQ and low-level resistance to clofazimine (CFZ).^8^,^9^ A growing amount of evidence has documented the emergence of acquired BDQ resistance among patients being treated with BDQ-containing regimens,^10^,^11^ and the rate of resistance-associated variants (RAVs) in *Rv0678* can be surprisingly high in BDQ-naïve patients.^12^

Uzbekistan is classified by the WHO as high MDR/RR-TB burden country, with an estimated incidence in 2019 of 3,200 cases (9.7/100,000 population).^13^,^14^ Karakalpakstan is an autonomous republic in the north-western part of Uzbekistan, with a population of 1.88 million in 2019. Médecins Sans Frontières (MSF) started working with the Ministry of Health (MOH) of Karakalpakstan to strengthen drug-susceptible TB (DS-TB) diagnosis and treatment in 1998,^15^ and a DOTS plus programme was initiated in 2003 to treat MDR/RR-TB patients.^16^ BDQ was included as part of the standard of care for MDR/RR-TB treatment in Karakalpakstan in 2015.

However, phenotypic drug susceptibility testing (pDST) for BDQ was only introduced in the Republican Center of Tuberculosis and Pulmonology in Karakalpakstan in October 2019 as a pilot activity, as there was a lack of global availability of BDQ pure substance and no international agreement on the critical concentration (CC) for performing DST. Stored samples from 2017 were tested retrospectively and the pDST for BDQ was fully incorporated into the routine testing algorithm for MDR/RR-TB patients in January 2020. A CC of 1.0 mg/L was used to determine susceptibility for BDQ using the proportion method on the BACTEC™ (Mycobacterial Growth Indicator Tube) MGIT™ 960 (BD, Franklin Lakes, NJ, USA), as recommended by the WHO.^17^ DST was repeated at the same CC for all samples with BDQ resistance before the results were released from the laboratory. Kone et al. conducted a multi-laboratory, multi-country study, validating the interim WHO CC of 1.0 mg/L for MGIT, and reported a sensitivity and specificity of respectively 100% (95% CI 97.6–100.0) and 99.8% (95% CI 98.8–100.0).^18^

The BD BACTEC MGIT 960 SIRE kit is used for pDST for rifampicin (RIF), isoniazid, pyrazinamide and ethambutol, and the test is performed at respectively 0.5, 1.0, 100 and 5.0 mg/L concentration. Sigma Aldrich^®^ (St Louis, MI, USA) is used for pDST for levofloxacin, moxifloxacin (MFX), linezolid and amikacin; a CC of 1.0 mg/L is used for levofloxacin, linezolid and amikacin, while for MFX, both concentrations, 0.25 and 1.0 mg/L are used. The pDST for CFZ was performed at a CC of 2.5 mg/L from 2019 to May 2022 because of a technical error in the laboratory. Hence, the results of CFZ DST were not considered in determining likely effective drugs in the treatment regimen of study patients due to the uncertainty around the negative result.^17^

Internal quality control is performed for each batch of specimen being processed using *H37Rv*-susceptible strain as positive control and sterile phosphate buffer as negative control.^19^ Batch testing is done for MGIT drug kits and second-line drugs using *H37Rv-*susceptible strain, together with one other resistant strain using the normal DST protocol.^20^ For external quality assessment (EQA), DST panels are provided by the WHO Supranational Reference Laboratory of Tuberculosis, Gauting, Germany, on a yearly basis. BDQ pure substance for DST is sourced from Janssen (Beerse, Belgium) via the National Institutes of Health Program (Bethesda, MD, USA).

This retrospective case series analyses routine programmatic data collected from the Karakalpakstan TB Programme. Treatment initiation and follow-up forms containing demographic characteristics, previous TB treatment history, underlying health problems and information about current TB treatment are recorded and encoded into an electronic database.

A previous study from Karakalpakstan highlighted the prevalence of acquired BDQ resistance in the region.^10^ This report will focus on the prevalence of BDQ resistance in patients with no history of BDQ exposure, i.e., primary resistance. We describe 12 patients with primary BDQ resistance and highlight potential associated risk factors. The purpose of this study is to improve understanding of primary BDQ resistance and share experience of managing these TB patients.

## METHOD

Data from records of all MDR/RR-TB patients treated from 2017 to 2022 were reviewed for evidence of treatment with a BDQ-containing regimen and pDST confirmed resistance to BDQ. Study-related data were extracted from laboratory and clinical databases into a password-protected MS Excel sheet (MicroSoft, Redmond, WA, USA). The file was only accessible to the study team. Hard copies of patient records are kept in a locked cabinet with limited access. Patients with primary BDQ resistance, defined as patients with MTB isolates exhibiting growth at MGIT 1.0 mg/L without prior exposure to a BDQ-containing regimen for more than 1 month, were included in the study population.

We used the standard WHO and Uzbekistan national definitions for TB treatment outcomes and culture conversion and reversion.^3^ Culture conversion was defined as two consecutive negative cultures after the first sputum sample, collected at least 30 days apart. We collected the following variables of interest: age, sex, presence of comorbidities, employment status, marital status, smoking status, alcohol intake, intravenous drug use (IVDU) history, migration history and history of previous exposure to BDQ and/or CFZ. Finally, we collected data on the initial treatment regimen, any changes in treatment regimen and culture result at baseline and each month until the end of treatment.

Information on close contacts and a list of likely effective TB drugs upon initiating treatment was gathered individually from patients’ medical records. The likelihood of effectiveness was judged on the basis of one or more of the following: phenotypic/genotypic DST, confirmed susceptibility in the presumed source case, no known resistance to another drug with cross resistance to the drug, rare use of the drug in a geographical area or setting (possibly supported by low drug resistance levels from surveillance activities) and no previous use of the drug in a regimen that failed to cure the individual patient.^3^ Baseline characteristics were described using frequencies and percentages for categorical variables, and medians with interquartile ranges (IQRs) for continuous variables.

The study fulfilled the exemption criteria set by the MSF’s independent Ethical Review Board (ERB) for an a posteriori analysis of routinely collected clinical data and thus did not require MSF ERB review.

## RESULTS

Overall, 2,247 patients were treated for MDR/RR-TB from January 2017 to March 2022 in Karakalpakstan. These cases were included in the initial screening for this study. Among these, 1,930 patients were tested for BDQ resistance phenotypically. BDQ resistance was found in 68 patients on the first test and confirmed in 64 on the second. Of 4 patients with unconfirmed resistance, 1 had BDQ exposure history and 3 had none. Thirty-five of the 64 confirmed BDQ-resistant patients did not have a baseline DST prior to initiating treatment, as they started treatment prior to the introduction of routine DST for BDQ, and hence could not be classified into primary or secondary resistance. We excluded 17 patients who were exposed to BDQ for more than 1 month prior to being diagnosed with BDQ resistance. The final study population included 12 patients who met the criteria for primary BDQ resistance (Figure).

**Figure:**
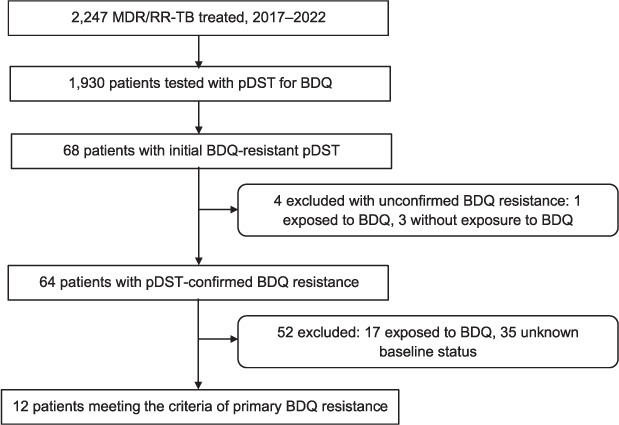
Study population. MDR/RR-TB = multidrug/rifampicin-resistant TB; pDST = phenotypic drug susceptibility testing; BDQ = bedaquiline.

The demographic characteristics and potential risk factors of the study population are presented in Table 1. The median age of the study population was 29 years (IQR 27–39). There were eight male patients, representing 66% (8/12) of the study population. Within this group, 5 (42%) had a history of TB contact, 2 (17%) had a history of travelling outside of the country and 2 (17%) were smokers. No patients reported a history of IVDU, incarceration or alcohol abuse. None of the patients were healthcare workers. Of 12 patients, 11 (92%) were unemployed at the time of detection of BDQ resistance.

**Table 1:** Demographic characteristics and potential risk factors of the study population

Patient characteristic		*n* (%)
Sex	Female	4 (33)
	Male	8 (67)
Age, years, median [IQR]	29 [27–39]	
Alcohol intake	None	10 (83)
	NA	2 (17)
Smoking status	Yes	2 (16.5)
	No	8 (67)
	NA	2 (16.5)
Employment status	Unemployed	7 (58)
	Employed	1 (8)
	Retired	1 (8)
	Disabled	1 (8)
	NA	2 (17)
Healthcare worker	No	10 (83)
	NA	2 (17)
History of IVDU	No	10 (83)
	NA	2 (17)
History of incarceration	No	10 (83)
	NA	2 (17)
History of travelling outside of the country	Yes	2 (16.5)
	No	8 (67)
	NA	2 (16.5)
History of TB contact	Yes	5 (42)
	No	5 (42)
	NA	2 (16)

IQR = interquartile range; NA = not available; IVDU = intravenous drug user.

Previous TB treatment history is presented in Table 2. Among the study population, 4 (33%) had been previously treated for DS-TB, 5 (42%) for MDR/RR-TB and 3 (25%) were treatment-naïve. Two patients (16.6%) were exposed to CFZ for more than 1 month before the BDQ resistance was detected.

**Table 2:** Previous TB treatment history of the study population

Study population		*n* (%)
Previous TB treatment history	DS-TB treatment	4 (33)
	MDR/RR-TB treatment	5 (42)
	TB-naïve	3 (25)
Exposure to clofazimine for more than 1 month in previous TB treatment episode	Yes	2 (16.5)
	No	8 (67)
	Unknown	2 (16.5)
Previous TB treatment outcome	TB-naïve	3 (25)
	Favourable outcome*	4 (33)
	Unfavourable outcome^†^	2 (16)
	Unknown	3 (25)

* Defined as TB treatment outcome that was cured or treatment completed.

† Includes failed treatment, loss to follow-up or death.

DS-TB = drug-susceptible TB; MDR/RR-TB = multidrug/rifampicin-resistant TB.

Patient D, whose DST result was susceptible to RIF, was on DS-TB treatment, and the remaining 11 patients were on individualised MDR/RR-TB treatment regimens (Table 3). The interim analysis found that Month 3 and Month 6 culture conversion was achieved in respectively 66% (8/12) and 50% of the study population. The most frequently prescribed medications for the study population were linezolid (92%), followed by CFZ (83%), cycloserine (75%), delamanid (75%) and MFX (58%). Of 12 patients, 3 (25%) had four or more likely effective drugs at the beginning of BDQ-resistant treatment.^3^

**Table 3:** Baseline DST, sputum culture, treatment regime and treatment outcome

Patient	Baseline resistance on DST*	Initial treatment regimen^†^	Sputum culture by month																				

			0	1	2	3	4	5	6	7	8	9	10	11	12	13	14	15	16	17	18	19	Outcome
A	HREZFqBdqCfz	BdqLzdCfzCsIpm/Cln	(+)	(+)	(+)	(+)																	Died
B	HREZFqBdq	BdqLzdCfzCsDlm	(+)	(+)	(−)	(−)	(−)	(−)	(−)	(−)	ND	(−)	(−)	(−)	(−)	(−)	(−)	(−)	(−)				Ongoing
C	HREZFqBdq	MfxBdqLzdCfzCsPth	(+)	(+)	(+)	(+)	(+)	(+)	(−)	(+)	(+)	(+)	(+)										Failure
D	Bdq	HREZ	(+)	(+)																			Completed
E	HERMfx(0.25)Bdq	Mfx^H^LzdCfzDlmIpm/Cln	(+)	(+)	(−)	(−)	(−)	(−)	(−)	(−)	(−)	(−)	(−)	(−)	(−)	(−)							LTFU
F	HERMfx(0.25)Bdq	Mfx^H^LzdCfzDlmIpm/Cln	(−)	(−)	(−)	(−)	(−)	(−)	(−)	(−)	(−)												Ongoing
G	HERMfx(0.25)Bdq	Mfx^H^LzdCfzCsDlmIpm/Cln	(+)	(−)	(−)	(−)	(−)	(−)	(−)	(−)	(−)	(−)	(−)										Ongoing
H	HREZFqBdq	BdqLzdCfzCsDlm	(+)	(+)	(−)	(−)	(−)	(−)	(−)	(−)	(−)	(−)	(−)	(−)	(−)	(−)	(−)	(−)	(−)	(−)	(−)	(−)	Cured
I	HRZBdq	BdqLzdCfzCsDlm	(+)	(+)	(−)	(+)	(−)	(−)	(−)	(−)	(−)	(−)	(−)	(−)	(−)	(−)	(−)	(−)	(−)	(−)	(−)		Ongoing
J	RFqBdq	MfxLzdCfzCSDLM	(+)	(+)	(−)	(−)	(−)																Ongoing
K	RFqBdq	LzdCfzCsDlmAmkIpm/Cln	(+)	(+)	(−)	(−)	(−)																Ongoing
L	RFqBdq	BdqLzdCfzCsDlm	(+)	(+)	(−)	(−)																	Ongoing

* The critical concentration of 2.5 mg/L was used for conducting phenotypic DST for Cfz, instead of 1.0 mg/L, which may have misclassified Cfz-resistant samples as susceptible.

^†^ Likely effective drugs underlined.

DST = drug susceptibility testing; H = isoniazid; R = rifampicin; E = ethambutol; Z = pyrazinamide; Fq = fluroquinolone; Bdq = bedaquiline; Cfz = clofazamine; Cs = cycloserine; Ipm/Cln = imipenem cilastatin (with added amoxicill-inclavulanate); (+) = positive; (−) = negative; Dlm = delamanid; Lzd = linezolid; Mfx = moxifloxacin; Pth = prothionamide; Mfx^H^ = high-dose Mfx; ND = not done; LTFU = loss to follow-up.

As of March 2022, three patients had an unsuccessful treatment outcome (3/12, 25%). Patient A died at Month 4 of TB treatment, Patient C was reported as treatment failure and Patient E was reported lost to follow-up (LTFU) after culture conversion for 12 consecutive months. Two patients had a successful outcome (17%, 2/12), one treatment completed and one cured. Seven patients were still on treatment (58%, 7/12). Eight patients (8/12, 66%) were culture-negative by Month 1 or Month 2 of treatment. Patient A and Patient C, who continued to be culture-positive at Month 3, had unfavourable treatment outcomes. The detailed culture status and treatment outcome of patients are given in Table 3.

## DISCUSSION

Key study findings are as follows: a large proportion of the cases (9/12, 75%) had previously been treated for TB, 83.3% (10/12) had concomitant fluoroquinolone (FQ) resistance and 25% (3/12) had poor treatment outcomes (1 died, 1 failed treatment, 1 LTFU). We also found that both patients who had not experienced culture conversion by Month 3 of treatment had an unfavourable outcome. This finding is consistent with previous studies, suggesting that culture conversion from Month 2 to Month 6 can be a prognostic marker for predicting treatment outcomes in MDR/RR-TB patients.^21^,^22^

Of the 12 study patients with primary BDQ resistance, 75% (9/12) had been previously treated for TB: four for DS-TB and five for DR-TB. Xu e al. found four BDQ and CFZ cross-resistant isolates in 90 (4.4%) clinical samples from previously treated pre-XDR-TB and XDR-TB patients, all of whom harboured *Rv0678* RAVs.^23^ In a study from the WHO Supranational Reference Centre for Mycobacteria in Borstel, Germany, three of 124 (2.4%) patients with MDR/RR-TB were found to have primary resistance to both BDQ and CFZ.^24^ In a third study of adult patients with DR-TB and HIV infection, 3/5 isolates with *Rv0678* RAVs had MICs at the top of the wild-type range.^25^ Three of the five (60%) had been previously treated for DR-TB (without BDQ and CFZ); this could be explored in future research to understand the relationship between prior exposure to TB drugs and the occurrence of BDQ-resistant TB.

To the best of our knowledge, the BDQ mono-resistance detected in our study is the first of its kind; a possible explanation for it could be that BDQ pDST was not routinely performed in RIF-susceptible patients. To note, Villellas et al. found a lower rate of *Rv0678* RAVs in non-MDR/RR-TB isolates (0.7%, 6/852), suggesting a role for natural resistance in BDQ resistance.^12^

Ten of the 12 study patients with primary BDQ resistance (83.3%) were FQ-resistant as well. This finding agrees with the Chinese and South African publications referenced above, where all BDQ-resistant isolates were also FQ-resistant.^23^,^25^

As of March 2022, we recorded treatment outcomes for 5/12 patients; however, three of these (3/12, 25%) had a poor outcome. One patient died after 3 months of treatment, one failed treatment and one was LTFU after culture conversion for 12 consecutive months. This finding is in accordance with the South African study in which 3/5 (60%) patients with baseline *Rv0678* mutations had an unsuccessful treatment outcome (two deaths, one LTFU) compared to 18.4% (16/82) without baseline *Rv0678* mutations.^25^ Villellas et al. did not report treatment outcomes but found no clear relationship between baseline BDQ minimum inhibitory concentrations (MICs) and culture conversion at the endpoint.^12^ On a more positive note, two of our BDQ-resistant patients (2/12, 17%) had a successful treatment outcome and seven (7/12, 58%) are currently stable on treatment.

Among MDR/RR-TB patients tested for BDQ resistance, defined as an MTB strain exhibiting growth at MGIT 1.0 mg/L, we found an overall prevalence of BDQ resistance of 3.3% (64/1,930, 95% confidence interval [CI] 2.6–4.2) and primary BDQ resistance of 0.6% (12/1,930, 95% CI 0.3–1.0). This compares to primary BDQ resistance of 2.4% (3/124) in the report by Andres et al. who used the same MIC cut-off value on MGIT.^24^ In three reports that used an MIC of ≥0.25 mg/L as the cut-off for resistance using the agar proportion method, the prevalence of primary BDQ resistance was 2.3% (8/347), 4.4% (4/90) and 3.1% (28/898).^12^,^23^,^26^

Of the 12 study patients, 3 (25%) were TB-naïve, suggesting the presence of primary transmission of BDQ-resistant strains in the community. The primary transmission of BDQ-resistant TB strains that has been documented in other similar high MDR-TB burden areas is a major public health concern,^27^ and can jeopardise the programmatic roll-out of a short-course 6-month treatment regime containing BDQ for MDR-TB patients. Baseline pDST for BDQ is recommended for initiating a BDQ-containing treatment regime for MDR-TB patients.

The limitations to our study are the small sample size and the use of retrospective, routinely collected programmatic data whose validity could not be ascertained. Nevertheless, patient medical records were registered by qualified physicians and entered into a database by an experienced team, which assures data quality. Genome sequencing was not available in our setting, and therefore, information about mutations and RAVs is not available. Finally, the technical error in performing pDST for CFZ at a CC of 2.5 mg/L may have led to false-susceptible results. CFZ could have been ineffective for some of the patients prescribed based on this DST result.

Primary resistance to BDQ at baseline should be carefully monitored by DR-TB treatment programmes in Central Asia and elsewhere. Where it has been identified, new DR-TB cases with a history of past TB treatment should be considered at risk of primary BDQ resistance and the introduction of gene sequencing technology should be contemplated. The treatment of BDQ-resistant DR-TB can be challenging, but a successful treatment outcome is achievable using an individualised treatment regimen based on drug resistance patterns and close monitoring of microbiology status.
